# Comparison of fetal heart rate baseline estimation by the cardiotocograph network and clinicians: a multidatabase retrospective assessment study

**DOI:** 10.3389/fcvm.2023.1059211

**Published:** 2023-08-09

**Authors:** Jieyun Bai, Xiuyu Pan, Yaosheng Lu, Mei Zhong, Huijin Wang, Zheng Zheng, Xiaohui Guo

**Affiliations:** ^1^ Guangdong Provincial Key Laboratory of Traditional Chinese Medicine Information Technology, Jinan University, Guangzhou, China; ^2^ College of Information Science and Technology, Jinan University, Guangzhou, China; ^3^ Auckland Bioengnieering Institute, The University of Auckland, Auckland, New Zeanland; ^4^ Department of Obstetrics and Gynecology, Guangzhou Women and Children's Medical Center, Preterm Birth Prevention and Treatment Research Unit, Guangzhou Medical University, Guangzhou, China; ^5^ Department of Obstetrics, NanFang Hospital of Southen Medical University, Guangzhou, China; ^6^ Department of Obstetrics, Shenzhen People's Hospital, Shenzhen, China

**Keywords:** artificial intelligence, deep learning, fetal heart rate, baseline, computer analysis

## Abstract

**Background:**

This study aims to compare the fetal heart rate (FHR) baseline predicted by the cardiotocograph network (CTGNet) with that estimated by clinicians.

**Material and methods:**

A total of 1,267 FHR recordings acquired with different electrical fetal monitors (EFM) were collected from five datasets: 84 FHR recordings acquired with F15 EFM (Edan, Shenzhen, China) from the Guangzhou Women and Children's Medical Center, 331 FHR recordings acquired with SRF618B5 EFM (Sanrui, Guangzhou, China), 234 FHR recordings acquired with F3 EFM (Lian-Med, Guangzhou, China) from the NanFang Hospital of Southen Medical University, 552 cardiotocographys (CTG) recorded using STAN S21 and S31 (Neoventa Medical, Mölndal, Sweden) and Avalon FM40 and FM50 (Philips Healthcare, Amsterdam, The Netherlands) from the University Hospital in Brno, Czech Republic, and 66 FHR recordings acquired using Avalon FM50 fetal monitor (Philips Healthcare, Amsterdam, The Netherlands) at St Vincent de Paul Hospital (Lille, France). Each FHR baseline was estimated by clinicians and CTGNet, respectively. And agreement between CTGNet and clinicians was evaluated using the kappa statistics, intra-class correlation coefficient, and the limits of agreement.

**Results:**

The number of differences <3 beats per minute (bpm), 3-5 bpm, 5–10 bpm and ≥10 bpm, is 64.88%, 15.94%, 14.44% and 4.74%, respectively. Kappa statistics and intra-class correlation coefficient are 0.873 and 0.969, respectively. Limits of agreement are −6.81 and 7.48 (mean difference: 0.36 and standard deviation: 3.64).

**Conclusion:**

An excellent agreement was found between CTGNet and clinicians in the baseline estimation from FHR recordings with different signal loss rates.

## 1. Introduction

Although deaths in children have declined substantially in the past 30 years, more than 5 million still die every year (1). Electronic fetal heart rate (FHR) monitoring was introduced to detect fetuses' pathological states as early as possible in the obstetrics practice in the late 1950s. However, the misinterpretation and ambiguity of FHR patterns may increase unnecessary interventions, such as operative deliveries and cesarean sections (2–4). Different guidelines over the past decades have recommended some modifications for interpreting FHR tracings, but beliefs in the etiology of basic FHR patterns (including the baseline, the variability, accelerations, decelerations, and sinusoidal patterns) have remained essentially unchanged (5, 6). In these FHR patterns, the baseline is a pre-requisite for evaluating the other patterns (7). Gynecologists and obstetricians usually estimated the baseline by visual analysis, but the unreliability of visual interpretation with a high degree of inter-and intra-observer variability is found (7–12). Therefore, computer-assisted analysis has been sought to mitigate the variability of visual explanation (13–17).

Several studies have evaluated the performance of different computer-assisted methods. For example, in 2016, Jezewski et al. evaluated 11 different algorithms using two inconsistency coefficients based on three properties (i.e., number, location and area) of accelerations/accelerations (18). They found that the algorithm of Arduini et al. (9, 19) outperforms other methods by achieving the lowest mean inconsistency coefficients on a private dataset with 41 FHR signals. This nonlinear filtering method is similar to the algorithm proposed by Mantel et al. (20). The difference is that Arduini's baseline is computed in 10 min windows with 5 min shift, whereas Mantel's baseline is calculated for the whole FHR tracing. Considering Mantel's method, Houzé de l'Aulnoit et al. further evaluated 11 newer algorithms by comparing the computed baselines with that estimated by clinicians on a dataset with 90 FHR signals (13). This study found that Lu and Wei's algorithm (14) achieves better results than other methods by achieving a new morphological analysis discriminant index (MADI) of 7.3%. Recently, a weighted median filter was proposed by Boudet et al. to compute the FHR baseline, and more agreement (represented by a MADI of 4.0%) with clinicians' consensus than Lu and Wei's method was shown on this dataset with 90 FHR recordings (15). Similar to Lu and Wei's method, an algorithm for the baseline estimation based on singular spectrum analysis and empirical mode decomposition was also proposed by Lu et al. (16) and evaluated on another public dataset with 552 FHR recordings. This method also was objectively evaluated on the dataset with 90 FHR recordings (13) by achieving a MADI of 15.6%. Unlike signal processing methods, the CTGNet based on deep learning was proposed in our previous study (21) and evaluated on a larger dataset with 234 FHR recordings. This method was compared with 12 signal processing methods and the lowest metrics (including the root-mean-squared difference between baselines and MADI) were obtained. These methodological studies illustrate the excellent performance of the CTGNet. However, its clinical application still requires a comparative study with large-scale multicenter data.

To evaluate the clinical usability of the CTGNet, we compare the FHR baseline predicted by the CTGNet with that estimated by clinicians using a large dataset with 1,267 FHR recordings acquired with fetal monitors of five device manufacturers.

## 2. Material and methods

### 2.1. Data source

**Figure 1:**
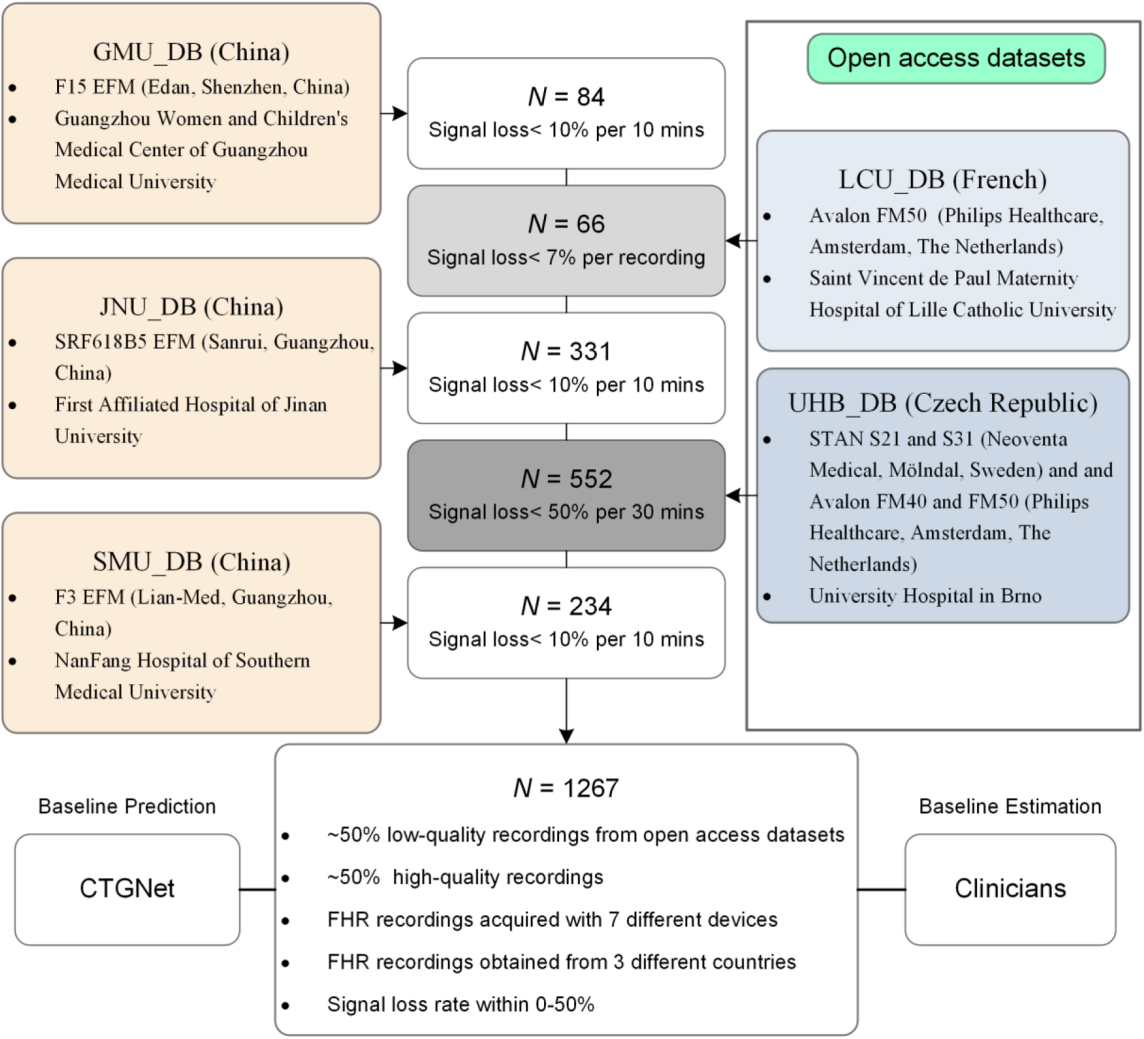
Flow chart of the FHR baseline estimation.

This prospective assessment study used 1,267 FHR recordings from 5 datasets covering the years 2011 to 2021: (1) an FHR dataset with 84 recordings collected from the Guangzhou Women and Children's Medical Center of Guangzhou Medical University (GMU_DB; May 2021 to July 2021); (2) a dataset with 331 CTG records of the First Affiliated Hospital of Jinan University (JNU_DB; January 2015 to December 2020); (3) a dataset with 234 CTG records collected from the NanFang Hospital of Southern Medical University (SMU_DB; January 2012 to December 2020); (4) the open-access database with 66 FHR recordings collected at Saint Vincent de Paul Maternity Hospital of Lille Catholic University (LCU_DB; February 1st, 2011, and December 31st, 2016) (13, 22, 23) and the open-access database with 552 CTG recordings collected at the obstetrics ward of the University Hospital in Brno (UHB_DB; April 2010 and August 2012) (24–26). In these datasets, the signal loss rate of FHR recordings from GMU_DB, JNU_DB and SMU_DB is <10% per 10 min, whereas those from GMU_DB and UHB_DB are <7% and <50%, respectively (Figure 1).

The Medical Ethics Committees of the Guangzhou Women and Children's Medical Center (273A01), the Jinan University (JNUKY-2022-018) and the NanFang Hospital of Southen Medical University (NFEC-2019-024) approved this retrospective study.

### 2.2. Devices

Fetal monitors used for acquiring FHR recordings include: F15 EFM (Edan, Shenzhen, China), SRF618B5 EFM (Sanrui, Guangzhou, China), F3 EFM (Lian-Med, Guangzhou, China), STAN S21 and S31 (Neoventa Medical, Mölndal, Sweden), and Avalon FM40 and FM50 (Philips Healthcare, Amsterdam, The Netherlands).

### 2.3. Methods

According to the baseline definition of the FIGO consensus guideline (5): (1) the baseline is estimated as the mean level of the most horizontal and less oscillatory FHR segments of 10 min; (2) It is necessary to review previous and subsequent 10 min sections to estimate the baseline in recordings with unstable FHR signals. Clinicians (Z.Z. and X.P.) with more than seven years of experience in CTG analysis independently assessed the baselines of FHR tracings. In order to obtain a consistent baseline, FHR recordings were re-evaluated when the differences between clinicians exceeded three bpm, and the baseline was determined as an average of these clinicians’ estimations when their difference was less than three bpm. A difference between the baseline estimated by clinicians and that predicted with the CTGNet were was then computed to evaluate their agreement.

### 2.4. Statistical analysis

For each FHR recording, baseline values estimated by CTGNet and the consensus of clinicians were attributed to 5 bpm classes (such as class 0: ≤100 bpm, class 1: 100 <baseline ≤105, and class 2: 105 <baseline ≤110) in the following manner (7): (1) when the baseline difference does not exceed five bpm, CTGNet's and clinicians' baselines are assigned to the same class according to their mean (e.g., if CTGNet's baseline value is 109 and clinicians' estimation is 113, both values are assigned to the class 110–115); (2) when the baseline difference exceeds five bpm, baseline values are assigned to their respective classes. Kappa and intra-class correlation (ICC) coefficient values (i.e., excellent agreement: >0.75, good agreement: 0.4–0.75 and poor agreement: <0.4) were calculated to evaluate agreement in the baseline estimation and 95% confidence intervals (95% CI) were computed for all results.

## 3. Results

Table 1 summarizes the comparisons of the baselines predicted by the CTGNet and those estimated by clinicians. In 99% of FHR recordings from GMU_DB, JNU_DB and SMU_DB, differences do not exceed 5 bpm, whereas the percentages of differences less than 5 bpm dropped to 58%–85% in FHR recordings from LCU_DB and UHB_DB. For the baseline difference ≥5 bpm, proportions are <0.5% for datasets from China (i.e., GMU_DB, JNU_DB and SMU_DB), 13.6% for LCU_DB, and 42.2% for UHB_DB, respectively. In general, the ratio of differences <5 bpm and ≥5 bpm is 4:1 among the 1,267 FHR recordings.

**Table 1:** Comparisons of the baselines predicted by the CTGNet and those estimated by clinicians.

	GMU_DB (*n *= 84)	JNU_DB (*n* = 331)	SMU_DB (*n* = 234)	LCU_DB (*n* = 66)	UHB_DB (*n* = 552)	All (*n* = 1,267)
Mean difference (bpm) (95% IC)	0.09 (−0.05 to 0.22)	−0.20 (−0.27 to −0.14)	0.88 (0.71 to 1.03)	0.72 (−0.99 to 2.41)	0.42 (0.02 to 0.83)	0.36 (0.14 to 0.54)
Standard deviation	0.63	0.58	1.28	7.05	4.83	3.64
Maximum differences (bpm)	2.81	3.43	16.52	48.88	18.86	48.88
Number of differences <3 bpm	83 (98.81%)	327 (98.79%)	227 (97.01%)	44 (66.67%)	141 (25.54%)	822 (64.88%)
Number of differences 3–5 bpm	1 (1.19%)	4 (1.21%)	6 (2.56%)	13 (19.70%)	178 (32.25%)	202 (15.94%)
Number of differences 5–10 bpm	0 (0.00%)	0 (0.00%)	0 (0.00%)	6 (9.09%)	177 (32.07%)	183 (14.44%)
Number of differences ≥10 bpm	0 (0.00%)	0 (0.00%)	1 (0.43%)	3 (4.55%)	56 (10.14%)	60 (4.74%)
Lower limit (95% IC)	−0.82 (−0.98 to −0.64)	−1.34 (−1.45 to −1.24)	−1.63 (−1.91 to −1.35)	−13.10 (−10.12 to −16.07)	−9.05 (−9.74 to −8.36)	−6.81 (−7.15 to −6.46)
Upper limit (95% IC)	0.93 (0.77 to 1.10)	0.93 (0.83 to 1.04)	3.39 (3.11 to 3.67)	14.52 (11.55 to 17.50)	9.90 (9.21 to 10.59)	7.48 (7.14 to 7.82)
Kappa (95% IC)	0.991 (0.974 to 1.008)	0.997 (0.992 to 1.001)	0.983 (0.966 to 1.000)	0.885 (0.808 to 0.963)	0.771 (0.745 to 0.797)	0.873 (0.857 to 0.889)
ICC (95% IC)	0.998 (0.997–0.999)	0.999 (0.999–1.000)	0.995 (0.993–0.996)	0.956 (0.928–0.973)	0.955 (0.947–0.962)	0.969 (0.966–0.972)

ICC, intra-class correlation coefficient; IC, confidence intervals.

According to Kappa values (i.e., excellent agreement: Kappa >0.75, good agreement: 0.4 ≤Kappa ≤0.75 and poor agreement: Kappa <0.4), agreement in the baseline estimation is excellent for different datasets. However, Kappa values for datasets from China (i.e., GMU_DB, JNU_DB and SMU_DB) are >0.98, whereas those for LCU_DB and UHB_DB are 0.885 and 0.771, respectively. For 1,267 FHR recordings, the Kappa value and the correlation coefficient between manual measurement and CTGNet calculation are 0.873 and 0.969, respectively.

**Figure 2:**
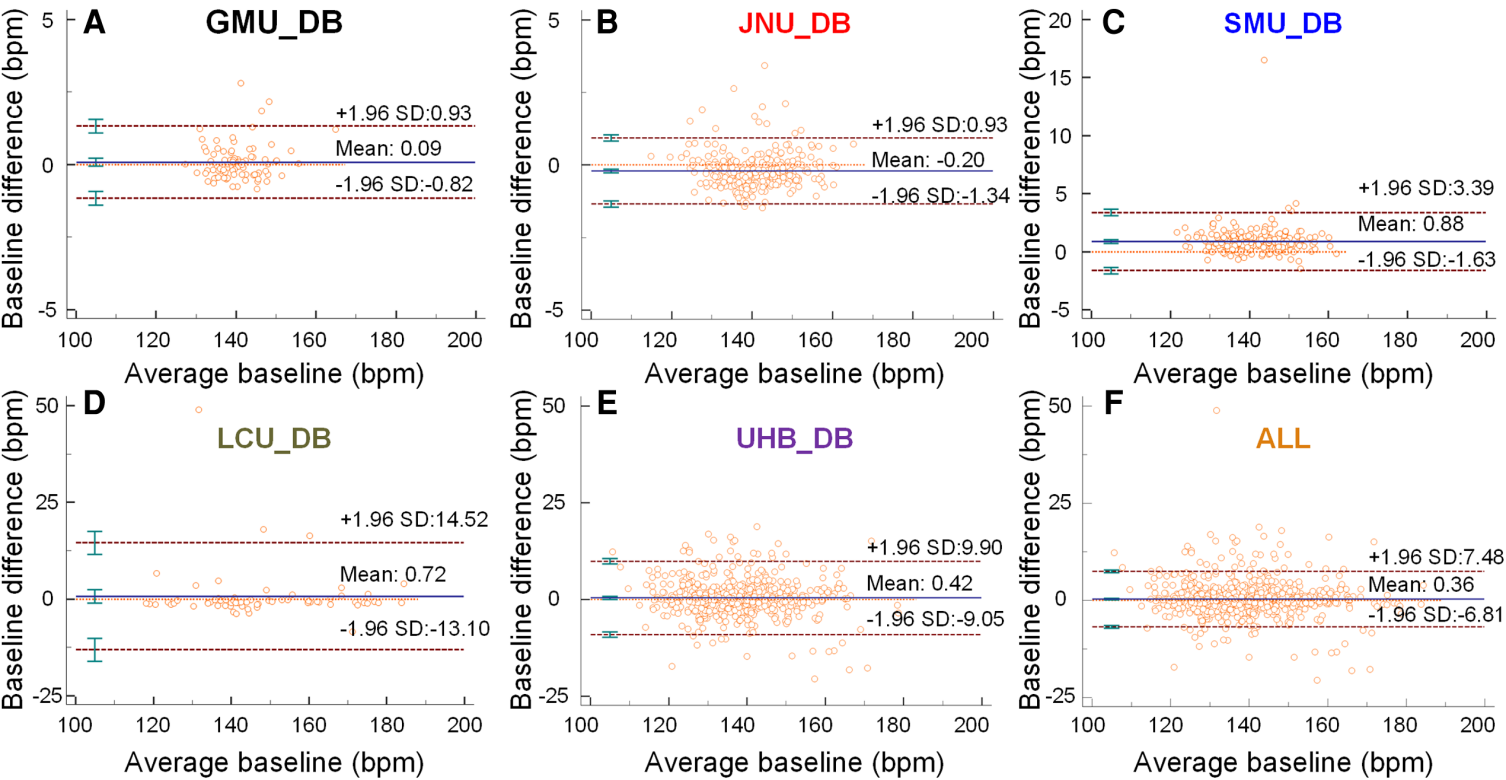
The baselines predicted by the CTGNet vs. those estimated by clinicians. (**A–F**) Bland-Altman plots for a comparison between automatic measurement and manual-measurement results about baselines on GMU_DB, JNU_DB, SMU_DB, LUC_DB, UHB_DB and ALL, respectively. Mean ±1.96 standard deviation (SD) is given.

Figures 2A–E are Bland-Altman plots demonstrating the interchangeability of the clinical measurement and the CTGNet for the baseline estimation. The mean differences are <1. Limits of agreement for GMU_DB, JNU_DB, SMU_DB, LUC_DB and UHB_DB, respectively, are −0.82/0.93, −1.34/0.93, −1.63/3.39, −13.10/14.52 and −9.05/9.90. Maximum differences for GMU_DB, JNU_DB, SMU_DB, LUC_DB and UHB_DB, are 2.81 bpm, 3.43 bpm, 16.52 bpm, 48.88 bpm and 18.86 bpm, respectively.

**Figure 3:**
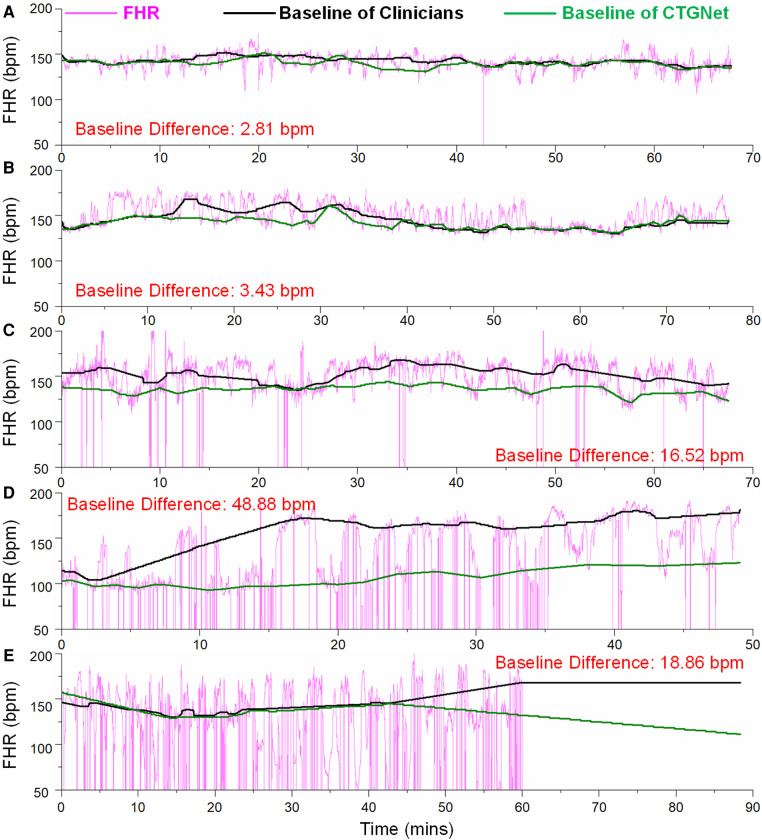
The most divergent examples in each database. (**A**) The maximum difference of 2.81 bpm on the FHR tracing from GMU_DB; (**B**) the maximum difference of 3.43 bpm on the FHR tracing from JNU_DB; (**C**) the maximum difference of 16.52 bpm on the FHR tracing from SMU_DB; (**D**) the maximum difference of 48.88 bpm on the FHR tracing from LUC_DB; (**E**) the maximum difference of 18.86 bpm on the FHR tracing from UHB_DB.

These most divergent examples in each database are shown in Figures 3A–F. It can be observed that the difference is large when the signal loss rate increases. Anyway, on the whole dataset including 1,267 FHR recordings, the mean difference, deviation and limits of agreement are 0.36, 3.64 and −6.81/7.48, respectively.

## 4. Discussion

Reliable FHR interpretation is the base of the fetal state assessment. The poor recognition performance of FHR patterns can propagate the error to subsequent steps, thereby decreasing classification accuracy. In all these FHR patterns, the baseline is a precondition for evaluating of the other patterns. Visual estimation of the FHR baseline is subject to inter-and intra-observer variability. Computer-assisted baseline estimation has been proposed as a promising way to reduce this variability. In order to evaluate the performance of our computer-assisted method (i.e., CTGNet), a comparison of FHR baseline estimation by the CTGNet and a consensus of clinicians presents in this study.

Baselines assigned by computer-assisted methods had been compared with those estimated by clinicians in several studies (7–12). In the studies of Arduini et al. and Ayres-de-Campo et al., limits of agreement (LoA) were no more than −6.45 and 7.07 in ≤150 FHR tracings. In addition, Kappa value and ICC coefficient also were used to evaluate agreement in baseline determination between a computer-assisted method and several experts in previous studies. Obtained Kappa values varied from 0.18 to 0.97, while the ICC coefficient was within the range of 0.83–0.98 (Table 2). All these results were obtained on several small datasets with ≤150 FHR tracings acquired with EFM from ≤3 different manufacturers. In the present study, a larger dataset with 1,267 FHR tracings acquired with EFM from 5 different manufacturers was used to evaluate agreement in baseline determination between the CTGNet and clinicians. This dataset included ∼50% high-quality tracings with a signal loss rate of <10% per 10 min and ∼50% low-quality recordings with a signal loss rate of <50% per 30 min. Kappa values were >0.98 for these high-quality tracings from GMU_DB, JNU_DB and SMU_DB, while the Kappa value was 0.771 for the lowest-quality FHR recordings (*n *= 552) from UHB_DB. Regardless, an excellent agreement was obtained on the whole dataset (*n *= 1,276). These results indicate possibilities for the clinical application of CTGNet in FHR baseline estimation.

**Table 2:** Studies of comparison between baselines assigned by computer-assisted methods and those estimated by clinicians.

Studies	Dataset	Signal loss	Devices	Metrics	Values
Arduini et al. (9)	Antepartum *n* = 34	–	Hewlett-Packard® 8,040A	LoA	−5.10 to 5.14
Todros et al. (8)	Antepartum *n* = 63	–	Hewlett-Packard® 8,040	Kappa	0.18–0.48
Mongelli et al. (10)	Intrapartum *n *= 60	–	–	ICC	0.83–0.97
Ayres-de-Campo (7)	Antepartum *n* = 149	<15%	Sonicaid® Meridien 800	Kappa	0.97 (0.90–1.00)
			Hewlett-Packard® M1350B	LoA	−3.22 to 5.22
			Hewlett-Packard® M1351–3	ICC	0.98 (0.97–0.99)
	Intrapartum *n *= 150	<15%	Sonicaid® Meridien 800	Kappa	0.87 (0.79–0.95)
			Hewlett-Packard® M1350B	LoA	−6.45 to 7.07
			Hewlett-Packard® M1351–3	ICC	0.95 (0.93–0.96)
Pinto et al. (11)	Intrapartum *n *= 40	–	STANs® 31	Kappa	0.83 (0.71–0.94)
Chen et al. (12)	Intrapartum *n *= 62	–	GE® Healthcare system	ICC	0.91 (0.88–0.94)
Ours	Intrapartum *n *= 1,267	<50%	Edan® F15, Sanrui® SRF618B5, Lian-Med® F3, STAN® S21 and S31, Avalon® FM40 and FM50	Kappa	0.873 (0.857–0.889)
				ICC	0.969 (0.966–0.972)
				LoA	−6.81 to 7.48

ICC, intra-class correlation coefficient; LoA, limits of agreement.

The high loss rate of FHR tracings severely affected the performance of CTGNet. In the present study, the Kappa value was reduced from 1.0 for the FHR recordings from JNU_DB with a low signal loss rate to 0.771 for the low-quality recordings from UHB_DB with a high signal loss rate. Although high-quality recordings with a signal loss of ≤20% are recommended by the FIGO guidelines to assess the FHR patterns (5), low-quality tracings with a mean signal loss of 28%–55% are shown in clinical practice (27, 28). For example, the mean signal loss of 13% and 30% were found during the first and the second stage of labor, respectively (29). Therefore, the CTGNet can be further trained on datasets with low-quality tracings to improve its robustness.

On these five datasets, we further compared the performance of our method (30) with existing signal processing methods. The method proposed by Mantal et al. achieved the best performance on the GMU_DB and JNU_DB datasets. Our method achieved the best results on the JNU_DB and LCU_DB datasets. The method proposed by Boudet et al. achieved the best results on the UHB_DB dataset. In the comprehensive evaluation of the five datasets, Boudet's method and our method ranked first and second respectively, and their baseline differences were both less than 3 bpm (Table 3).

**Table 3:** The baseline difference in bpm of different methods on five datasets.

	GMU_DB (*n *= 84)	JNU_DB (*n *= 331)	SMU_DB (*n *= 234)	LCU_DB (*n *= 66)	UHB_DB (*n *= 552)	All (*n* = 1,267)^a^
Houze de L’ Auinoit et al.	3.45	3.30	3.97	8.64	9.02	5.676
Mantel et al.	1.66	1.92	1.79	8.84	6.95	4.232
Mongelli et al.	2.52	2.44	2.86	5.47	7.13	4.084
Ayres-de-Campos et al.	2.80	3.06	2.96	8.11	9.68	5.322
Taylor et al.	3.44	3.41	3.82	8.69	11.71	6.214
Cazares et al.	3.39	3.78	3.49	7.72	8.58	5.392
Jimenez et al.	4.44	4.06	4.49	8.14	12.64	6.754
Pardey et al.	3.00	3.04	3.36	6.69	7.76	4.770
Lu and Wei	2.30	2.48	2.48	5.36	6.89	3.902
Maeda et al.	3.56	3.44	3.64	7.27	11.55	5.892
Wróbel et al.	3.75	3.23	4.70	6.23	8.00	5.182
Boudet et al.	2.04	1.92	2.30	4.42	7.23	3.582
Ours	2.02	1.70	2.22	3.71	5.17	2.964

^a^ Denotes the average result on five datasets.

## 5. Conclusions

The CTGNet for the FHR baseline estimation provided an excellent agreement with clinicians. However, this occurs in FHR recordings with low and medium signal loss rates. In the future, the CTGNet can be further improved by training it with more low-quality tracings.

## Data Availability

The original contributions presented in the study are included in the article, further inquiries can be directed to the corresponding authors.
